# Reward History and Statistical Learning Independently Impact Attention Search: An ERP Study

**DOI:** 10.3390/brainsci14090874

**Published:** 2024-08-29

**Authors:** Guang Zhao, Rongtao Wu, Huijun Wang, Jiahuan Chen, Shiyi Li, Qiang Wang, Hong-Jin Sun

**Affiliations:** 1 Faculty of Psychology, Tianjin Normal University, Tianjin 300387, China; wurongtao0331@163.com (R.W.); 15340705354@163.com (H.W.); cjh115660@163.com (J.C.); elevenny@hotmail.com (S.L.); wangqiang113@gmail.com (Q.W.); 2 Department of Psychology, Neuroscience and Behaviour, McMaster University, Hamilton, ON L8S 4K1, Canada; sunhong@mcmaster.ca

**Keywords:** reward history, statistical learning, attention control, attention priority map, independence

## Abstract

Selection history is widely accepted as a vital source in attention control. Reward history indicates that a learned association captures attention even when the reward is no longer presented, while statistical learning indicates that a learned probability exerts its influence on attentional control (facilitation or inhibition). Existing research has shown that the effects of the reward history and statistical learning are additive, suggesting that these two components influence attention priority through different pathways. In the current study, leveraging the temporal resolution advantages of EEG, we explored whether these two components represent independent sources of attentional bias. The results revealed faster responses to the target at the high-probability location compared to low-probability locations. Both the target and distractor at high-probability locations elicited larger early Pd (50–150 ms) and Pd (150–250 ms) components. The reward distractor slowed the target search and elicited a larger N2pc (180–350 ms). Further, no interaction between statistical learning and the reward history was observed in RTs or N2pc. The different types of temporal progression in attention control indicate that statistical learning and the reward history independently modulate the attention priority map.

## 1. Introduction

When encountering diversity in daily life, where multiple stimuli are presented simultaneously, it is meaningful to prioritize important stimuli and inhibit non-essential ones. Previously, it was believed that individuals could prioritize specific stimuli based on the current task goals, known as goal-directed attentional control [1]. Similarly, even in the absence of specific task requirements, individuals are susceptible to the attentional capture of salient stimuli in the environment, known as stimulus-driven attentional control [1]. However, this traditional theoretical dichotomy fails to explain the impact of experiential learning on attentional selection, especially when such learning is neither task-relevant nor physically salient. Recent research indicates that selective attention is also dictated by a new attention mechanism based on mechanisms that independently regulate and ultimately feed into an integrated priority map of attention [2]. Selection history emphasizes that lingering biases from previous selection experience play a key role in attentional selection and this comprises more than one distinct underlying mechanism of attention control [3]. Learned experience can be manifested in empirical probabilities of stimulus occurrence or associations with rewards, referred to as statistical learning or reward history, respectively [4,5,6,7,8,9].

Statistical learning benefits from the way in which our visual system is tuned to regularities within the environment, leading to the deployment of attention allocated to relevant regularities. Exposure to regularities regarding a distractor location induces spatially selective suppression [8]. In research using the additional singleton task, observers were asked to search for a specified shape among uniform shapes (e.g., a circle among diamonds) while ignoring an irrelevant color singleton. Crucially, instead of being distributed evenly across the display, the distractor singleton appeared much more frequently at one specific location (high-probability location) compared to all other locations combined (low-probability locations). For this high-probability location, it was observed that both the attentional capture by distractors and the efficiency of selecting the target were diminished, as the individuals learned to inhibit the location where the singleton distractor frequently occurred [7,8,9]. Theeuwes [10] proposed that spatial statistical learning functions by constantly modifying weights within a presumed spatial priority map, which dynamically manages the deployment of covert attention at any given moment. Locations that previously contained relevant information are upregulated, while locations with a higher likelihood of containing distractions are downregulated [11,12,13]. Observing that the influence of location probabilities on attentional selection resembles the learning of habits in several ways, such as being incidental, gradual, and unaffected by the working memory load [14,15], the mechanism by which statistical learning guides attention was ultimately thought to be habit-based [16]. Importantly, in the extensive studies on statistical learning, the participants were even unaware of the location probability manipulation. Thus, it is considered to be an implicit process that integrates statistical patterns from the input, functioning without conscious intention and beyond explicit awareness [17].

Regarding the reward history, a reward alters how a stimulus is represented by forming repetitive associations, while establishing associations between neutral stimuli and rewards enhances the attentional capture of these neutral stimuli, regardless of their relevance to tasks [5,6,18]. Anderson [5] started an experiment with a training phase where the target was defined in specified colors (red or green). Correct responses to one specific color earned a higher reward, while the other color was linked to a smaller reward. During the test phase, these colors were detached from the target but corresponded to an unrelated distractor, although no reward was provided. The results in the test phase revealed an enduring effect of reward learning on attentional priority: stimuli previously associated with rewards in the training phase captured attention when presented as irrelevant distractors, without the need for further reward learning. Substantial evidence points to Pavlovian associative learning as the mechanism by which previously reward-associated and/or aversively conditioned stimuli gain attentional priority, even without feedback [19,20]. This association affects how rewards alter the relevance of a stimulus, prioritizing its attentional processing [21]. A reward-based selection history adjusts the salience of the search array to allocate attentional resources [18,21].

This emphasis on experiential learning effects, as found in previous research, suggests that statistical learning and the reward history may independently influence target searching. The way in which statistical learning guides attention is habit-based, contingent on the repetition of past orienting behavior and driven primarily by reinforcement learning mechanisms [16,22]. A reward history develops through associative learning, establishing a predictive relationship between a stimulus and reward. Therefore, even after reward feedback is discontinued, such stimuli capable of predicting outcomes can still evoke automatic orienting responses. The reward history enhances the salience of reward stimuli, and, together with the weighting adjustment of corresponding spatial locations/features by statistical learning, feeds into the priority map independently. Anderson [23] used variants of an additional singleton task to investigate whether the statistical learning and the reward history, although established separately, function as independent sources of attentional mechanisms. During the training session, a color distractor singleton frequently appeared in a spatial location (66.7%). In half of the trials, the distractor was absent, the target randomly appeared in two different colors associated with varied rewards, and correct responses received the corresponding reward feedback. In the test phase, the color distractor singleton was the previously reward-related color, randomly appearing in all positions without any reward feedback. The results in the test phase revealed enduring synthesis regarding both the suppression effect of high-probability locations and the attentional capture effects of the reward, without a significant interaction between statistical learning and the reward history. The separation of these two components of the selection history indicates that they are independent sources in biasing attention. Statistical learning induces the proactive suppression of distractors at high-probability locations, downregulating the weight of spatial attentional priority for the location [12]. Meanwhile, reward-driven capture upregulates the physical salience of corresponding items [24]. Neural evidence also supports the notion that these two learning mechanisms influence attention priority through different paths. In the field of statistical learning, it is known that the medial temporal lobe (MTL), particularly the hippocampus, plays a crucial role in the rapid extraction of regularities from the environment [25,26,27]. Reward association leads to increased priority for the stimuli through the dopamine neurons in the midbrain and ventral striatum, ultimately reflected in the visual cortex [28]. Taken together, these findings indicate that the attention-enhancing effects of value and the attention-suppressing effects of statistical location learning independently influence overall attentional priority.

The current study aims to verify whether statistical learning and the reward history operate as independent sources of attentional bias during a visual search and to gain a deeper understanding of the neural basis of these learning effects in attentional mechanisms. Previous research on distractor statistical learning found that a Pd component, rather than the N2pc component, was elicited when distractors appeared at the high-probability location. This suggests the preemptive suppression of attentional allocation to the high-probability location and a reduction in its weight during the early processing of stimulus presentation [29,30,31,32,33,34,35,36,37,38]. In contrast to the reward history, a larger N2pc was observed when the distractor was associated with rewards compared to the non-reward condition, indicating greater attentional capture for reward-related distractors [39,40,41]. This suggests the possibility of reward-driven facilitation mechanisms, where stimuli associated with rewards become more prominent in the visual system.

However, previous studies have mainly focused on the effects of statistical learning and the reward history on attention searching individually. For example, Anderson [5] explored the reward history with contextually irrelevant stimuli, demonstrating that reward-related features can capture attention regardless of their relevance to the task or whether reward feedback is no longer provided. EEG studies using a similar task have shown a larger N2pc component [41]. Wang [8] explored the sustained inhibition effect resulting from distractor location statistical learning, indicating that both the target and distractor showed attentional suppression at high-probability locations. This was reflected in the EEG data, with a larger Pd component and a smaller N2pc [38]. Although this facilitates a better understanding of the effects of different types of experience learning within the selection history, it does not address whether these components rely on the same attentional mechanisms. To the best of our knowledge, few studies have explored statistical learning and the reward history in competing for attentional resources simultaneously [23,42,43]. Moreover, the conclusions are merely on behavioral results, and the studies do not provide an in-depth analysis of the two components in relation to different cognitive activities.

Considering that EEG provides the detailed temporal dynamics of cognitive processes, we hope to leverage the high temporal resolution of EEG to analyze whether these two components can independently influence attention over time in a dynamic manner. Statistical learning leads to the proactive suppression of specific locations, and the reward history, which enhances capture, impacts the attentional priority map in the opposite direction. In this study, statistical learning and the reward history with distractors are consistently associated throughout the experiment [43], and different reward distractors share a common high-probability location. This task paradigm excludes the possibility that partial independence may have resulted from separately establishing selection histories, as discussed by Anderson [23]. By this association manipulation between statistical learning and the reward history, we expect to observe the independent influences of the selection history both in the behavioral and EEG results. Specifically, the reward distractors will attract more attention compared to the non-reward condition, and this attentional capture will diminish when the distractors appear at the high-probability location rather than in other locations, indicating a form of location-specific suppression. Furthermore, there will be no interaction between statistical learning and the reward history.

## 2. Materials and Methods

### 2.1. Participants

Twenty-four participants were recruited from Tianjin Normal University (mean age 21.9 years, SD = 2.14, age range 18 to 26 years; 8 males). Participants were compensated at a rate of RMB 60 per hour and points were earned. The sample size was determined based on the G*power (with default settings), which revealed that this would give power of 0.80 to detect medium within-subjects effects (d_z_ = 0.6). Previous research has demonstrated medium effect sizes (d_z_ = 0.6) regarding the impact of rewards on attention [44]. Additionally, large effect sizes (d_z_ = 0.69–2.15) have been found for the impact of statistical learning regarding the distractor location [7,45,46].

All participants were right-handed, with normal or corrected-to-normal vision and (self-reported) normal color vision. None of the participants had taken part in similar experiments within the past six months, and there was no history of any physical illness or mental disorder. The research protocol was approved by the Ethics Committee of Tianjin Normal University. Informed consent was obtained from all participants before the experiment.

### 2.2. Instrumentation and Stimulation

Participants were instructed to sit in a soundproof and appropriately lit, shielded room, positioned approximately 60 cm from the computer monitor, and keep their head and body in a fixed position as much as possible and respond using the “F” or “J” keys on the keyboard. The experiment was programmed using E-prime 3.0, with stimuli presented on a 19-inch monitor at a resolution of 1024 × 768 pixels and a refresh rate of 60 Hz.

**Figure 1:**
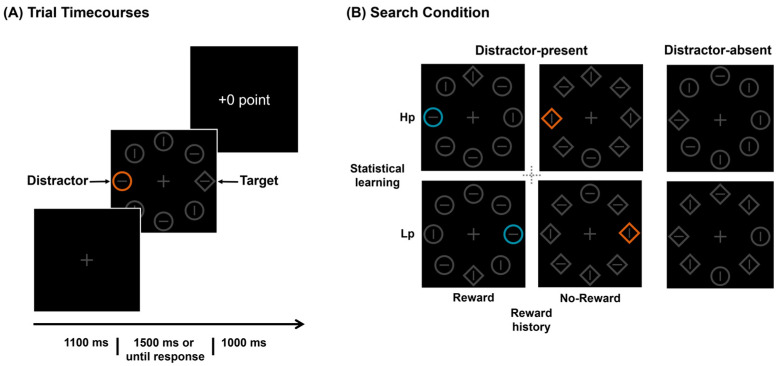
(**A**) Experimental flowchart. Participants judge lines within a single shape (a diamond among circles or a circle among diamonds). (**B**) Search conditions. The search array may contain a color singleton (distractor) or not, with the colors being blue or orange. Statistical learning: distractors are more likely to appear on the horizontal side (e.g., the left side as shown), referred to as the high-probability location (Hp), while the probability of appearance is equal in the vertical direction and the other horizontal side (e.g., the right side as shown) is referred to as a low-probability location (Lp). Reward history: when the distractor is blue, the correct response to the target will incur feedback of “+100 points”; when it is orange, the feedback is “+0 points”, balanced across the participants.

The experiment employed an additional singleton paradigm. As illustrated in Figure 1A, the search display consisted of eight items evenly distributed on an imaginary circle with a radius of 140 pixels from the fixation point on a black background. The target was a circle among diamonds or vice versa. All stimuli were uniformly gray (RGB: 70,70,70). In 2/3 of the trials, a non-target item appeared randomly in either blue (RGB: 37,141,165) or orange (RGB: 193,95,30), serving as a color distractor singleton. All items were sized to 72 × 72 pixels, with a horizontal or vertical line inside. The lines were randomly distributed and balanced among the eight stimuli. The target and the color singleton appeared only in the horizontal or vertical positions of the imaginary circle, while the remaining positions displayed neutral elements. The colors were chosen with the intention that the color distractor singleton would have higher luminance than the other (grey) display items, thus enhancing the distractor’s physical salience, as in previous work [44,47,48].

### 2.3. Design and Process

Each trial (see Figure 1A) began with a 1100 ms fixation display, followed by the search display. Participants were required to quickly and accurately judge the orientation of the lines inside the target (pressing “F” for horizontal or “J” for vertical). Before the formal experiment, each participant practiced 36 trials until their accuracy exceeded 75%. Participants were required to complete a total of 780 trials in the formal experiment, evenly divided into 10 blocks. On distractor-absent trials (240 trials), targets appeared in each of four locations equally often. On distractor-present trials (540 trials), the distractor was more likely (66.7%) to appear either on the left or right side in a horizontal position (high-probability position), counterbalanced across the participants. When the distractor was blue/orange, a correct response to the target would earn “+100/0 points” feedback. Distractors associated with different rewards appeared equally in high-probability positions and were evenly distributed among the remaining low-probability positions. For each participant, the target appeared with equal probability across four positions. In the distractor-present trials, the overall frequency of reward/no-reward conditions was balanced, with each condition occurring 180 times at the high-probability location. In the remaining three low-probability locations, the distribution was uniform, with the reward versus no-reward condition occurring 30 times each at any given low-probability location. Based on the above design, we divided the experiment into five conditions (see Figure 1B): (1) distractor-absent trials; (2) distractors at high-probability locations with rewards; (3) distractors at high-probability locations with no reward; (4) distractors at low-probability locations with rewards; (5) distractors at low-probability locations with no reward. After 1500 ms or until a response was given, the feedback was displayed.

## 3. Data Analysis

RTs faster than 200 ms or exceeding 2.5 SDs of the mean for the given 24 participants were excluded.

The computer recorded the brain activity from 64 scalp locations at a rate of 1 kHz, with electrodes placed on an elastic cap according to the international 10–20 system [49]. During the experiment, the impedance of all electrodes was maintained below 5 kΩ. The Cz electrode was used as the online reference, and, offline, EEG data were re-referenced using both mastoids. MATLAB with the EEGLAB 2022 toolbox [50] was used for EEG data analysis, employing a bandpass filter from 0.1 to 30 Hz [39,41]. After filtering, data from −200 ms to 1200 ms were extracted for each event, with −200 ms used for baseline correction. Continuous EEG data were manually inspected to eliminate obvious noise. Subsequently, independent component analysis (ICA) and visual identification were used to remove artifacts caused by blinks and eye movements [51]. Poor channels and trials with large artifacts, such as any absolute amplitude exceeding ±100 μV, were removed before further ERP averaging.

The ERP components primarily included the early Pd, Pd, and N2pc. The time windows were selected based on previous studies [31,38,51,52,53,54]. The early Pd epoch was defined as ±20 ms from the most positive peak between 50 and 150 ms [31,38]. The Pd epoch was defined as ±20 ms from the most positive peak between 150 and 250 ms [38,54]. The N2pc epoch was defined as ±20 ms from the most negative peak between 180 and 350 ms [38,51,52,53]. The averaged peak amplitude for these time windows was calculated separately for each participant and each condition [38]. Early Pd, Pd, and N2pc were all difference waves, calculated by subtracting the ipsilateral temporal lobe electrode from the contralateral temporal lobe electrode to determine the magnitude of the difference. PO7/8 electrodes were selected for this data analysis [38]. For the analysis of the ERP results for all participants, we only examined trials where the target or distractor was horizontal while the other one was vertical.

## 4. Results

### 4.1. Behavioral Results

**Figure 2:**
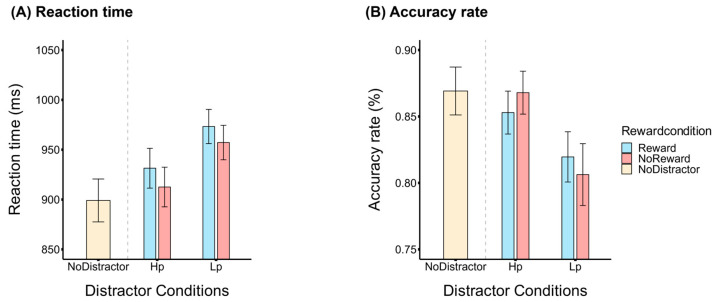
Behavioral results. (**A**) Mean reaction times (RTs) across all conditions. (**B**) Mean accuracy rate (ACC) across all conditions. The error bar represents the standard error.

We first analyze the trials with the distractor-present condition. For the response times, we conducted a 2 (location probability: high probability/low probability) × 2 (reward condition: reward/no reward) repeated-measures analysis of variance (ANOVA). As shown in Figure 2A, the results showed that the main effect of the distractor location probability was significant, *F* (1,23) = 41.44, *p* < 0.001, *η_p_*^2^ = 0.643, with faster responses for distractors present at the high-probability location than those at the low-probability locations. The main effect of the reward condition was also significant, *F* (1,23) = 11.96, *p* = 0.002, *η_p_*^2^ = 0.342, with slower responses for the reward than no-reward condition. However, the interaction was not significant, *F* (1,23) = 0.056, *p* = 0.815, *η_p_*^2^ = 0.002, suggesting an independent effect between the location probability and reward when distractors were present. The accuracy revealed the same pattern, as shown in Figure 2B. There was a significant main effect of the distractor location probability, *F* (1,23) = 24.16, *p* < 0.001, *η_p_*^2^ = 0.512. This indicated higher accuracy when distractors appeared at a high-probability location. Neither the main effect of the reward condition nor the interaction was significant, *Fs* ≤ 0.007, *ps* ≥ 0.099. Both the accuracy and response times suggested that there was no time and accuracy trade-off.

To examine the attention-capturing effect of salient distractors, we also compared the trials with the distractor-present and distractor-absent conditions. In the pair-wise *t*-tests between the distractor-present and distractor-absent conditions, when the distractor was absent, the participants searched for the targets significantly faster compared to when the distractors were present, *t* (23) = −7.40, *p* < 0.001, *Cohen’s d* = −1.51. The accuracy was also significantly higher in the distractor-absent trials, *t* (23) = 5.1, *p* < 0.001, *Cohen’s d* = 1.04. These findings indicate that the presence of distractors compromises the target response to some extent.

### 4.2. EEG Results

#### 4.2.1. Statistical Learning-Related Early Pd, Pd, and N2pc

**Figure 3:**
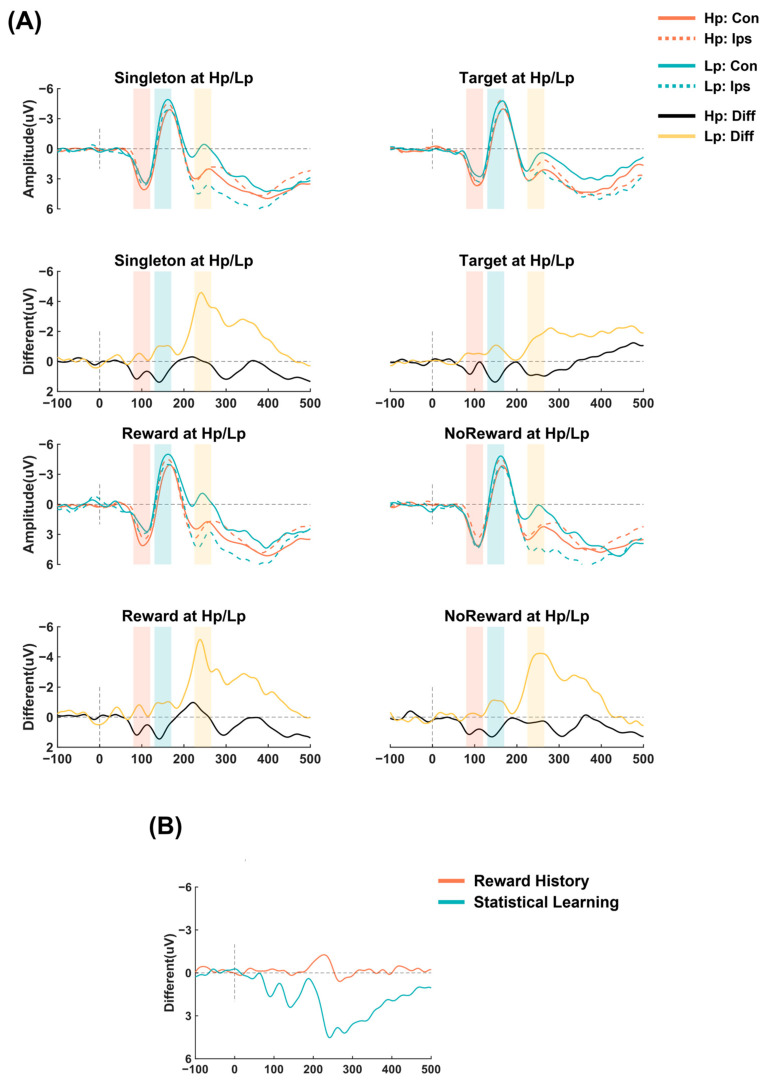
ERP results. (**A**) Top and middle lower images: The orange/blue lines represent the original ERP waves when the stimulus is at a high-/low-probability location, respectively. Solid/dashed lines represent contralateral/ipsilateral channels, respectively. Middle upper and lower images: The black/yellow lines represent the difference waves when the stimulus is at a high-/low-probability location, respectively. The red/blue/yellow shaded areas in all images represent the time windows for early Pd, Pd, and N2pc, respectively. (**B**) The main effect ERP difference waves for statistical learning and reward history, with orange/blue representing reward history/statistical learning, respectively. For the main effect of the reward history, the difference was calculated as the average amplitude of the reward condition minus the average amplitude of the non-reward condition. For statistical learning, the difference was calculated as the average amplitude under the high-probability condition minus the average amplitude under the low-probability condition.

Figure 3A depicts the ERPs obtained for different types of stimuli that appeared in various probability conditions. During the early Pd time window, shaded in red in the top panel, stimuli at a high probability elicited greater positive activation than those at a low probability. To test whether the early Pd was generated when the distractor or target was presented at the high-probability location compared to the low-probability locations, we conducted a two-way ANOVA on the different amplitudes of the early Pd with 2 (location probability: high probability/low probability) × 2 (stimulus type: distractor/target). This indicated a significant main effect of the location probability, *F* (1,23) = 24.37, *p* < 0.001, *η_p_*^2^ = 0.515, with a larger early Pd component generated when the distractor was in the high-probability location, indicating a suppression effect. Neither the main effect of the stimulus type nor the interaction was significant, *Fs* ≤ 1.17, *ps* ≥ 0.291, reflecting an equivalent level of suppression for both the target and the distractor, and the enhanced attentional inhibition of the distractors at the high-probability location was consistent with that of the target.

As shown in the blue-shaded areas in the top and upper-middle images of Figure 3A, during the Pd time window, stimuli at a high probability elicited greater positive activation. To provide a more comprehensive explanation of the learning suppression effect on the distractor’s location, we further examined the different ERP amplitudes of the Pd via a two-way ANOVA with 2 (location probability: high probability/low probability) × 2 (stimulus type: distractor/target). This indicated a significant main effect of the location, *F* (1,23) = 8.36, *p* = 0.008, *η_p_*^2^ = 0.267, with a greater Pd elicited when the stimuli were present at the high-probability location, suggesting the attentional suppression effect of this location. Neither the main effect of the stimulus type nor the interaction was significant, *Fs* ≤ 0.76, *ps* ≥ 0.391, indicating that the attentional suppression effects of the target were similar to those of the distractor and the same extent of enhanced attentional suppression of the target as for the distractor.

During the N2pc time window, in the yellow-shaded areas in the top and upper-middle images (Figure 3A), stimuli at a high probability elicited smaller negative activation than those at a low probability, and the distractor elicited greater negative activation than the target at low-probability locations compared to high-probability locations. To determine whether the target or distractor at high- and low-probability positions elicited attentional selection, we conducted a 2 (location probability: high probability/low probability) × 2 (stimulus type: distractor/target) analysis of the different ERP amplitudes of the N2pc. This indicated that the main effect of the location was significant, *F* (1,23) = 6.05, *p* = 0.022, *η_p_*^2^ = 0.208, where the stimuli at the low-probability locations elicited a larger N2pc, reflecting greater attentional capture. The main effect of the stimulus type was significant, *F* (1,23) = 9.84, *p* = 0.005, *η_p_*^2^ = 0.30, as the distractors elicited a larger N2pc, suggesting the larger attentional capture of distractors. There was a significant interaction, suggesting that the effect of the stimulus type was more pronounced at low-probability locations (*M*_distractor_ = −3.97 ± 3.07 μV, *M*_target_ = −2.22 ± 3.05 μV) than at high-probability locations (*M*_distractor_ = −0.89 ± 2.78 μV, *M*_target_ = −0.48 ± 2.83 μV), *F* (1,23) = 4.45, *p* = 0.046, *η_p_*^2^ = 0.162.

#### 4.2.2. Reward History and Its Relationship with Statistical Learning

As shown in the yellow-shaded areas in the lower-middle and lower images of Figure 3A, during the N2pc time window, reward distractors elicited greater negative activation, and stimuli at low-probability locations elicited greater negative activation. To investigate the attentional capture induced by the different reward conditions at high- and low-probability locations, we conducted a 2 (reward condition: reward/no reward) ×2 (location probability: high/low probability) repeated-measures ANOVA. The results showed a significant main effect of the reward condition, *F* (1,23) = 5.3, *p* = 0.031, *η_p_^2^* = 0.187, as a larger N2pc was elicited by the reward-related distractor, suggesting more attentional capture by reward distractors compared to no-reward ones. This indicated a significant main effect of the location probability, *F* (1,23) = 8.96, *p* = 0.007, *η_p_*^2^ = 0.28, where distractors that appeared at low-probability locations compared to high-probability locations generated greater N2pc, reflecting larger attention capture. Notably, the interaction between the reward condition and location probability was not significant, *F* (1,23) = 1.20, *p* = 0.285, *η_p_*^2^ = 0.05, consistent with the behavioral results, indicating that the attentional suppression effects of distractors high- and low-probability locations were independent of the attentional enhancement effects induced by the rewards.

## 5. Discussion

The current research aimed to investigate how distractor statistical learning and reward history influence visual searching under competing attentional demands. We used an additional singleton task, combining spatial statistical learning and a feature-related reward history with a color singleton. The behavioral results showed that when a distractor was present at a high-probability location, the response to the target was faster compared to those at low-probability locations. Reward distractors slowed the responses to the target compared to non-reward distractors. Further, no interactions were obtained in both reaction times and accuracies. The EEG recordings supported these findings. Both the target and the distractor presented at the high-probability location elicited stronger early Pd and Pd components. The distractor and the target at low-probability locations induced larger N2pcs than that in the high probability location. Additionally, reward distractors evoked a larger N2pc compared to non-reward distractors. There was no interaction between the location probability and reward condition regarding N2pc.

The current study provides evidence for the independent cognitive processing of statistical learning and the reward history under conditions where they compete for attentional resources. First, the impact of location-based statistical learning and the feature-based reward history on attention reflects different temporal processes. Our results showed that distractor statistical learning can guide attentional searching at an earlier time course compared to the reward history. Statistical learning induced earlier ERP components, including the early Pd. We firstly utilized the high temporal resolution of EEG to dynamically observe how statistical learning and the reward history each influence cognitive processes. These results are supported by evidence from previous studies that investigated statistical learning and reward history [19,38,39,40,41,55,56]. Statistical learning has shown that distractors at high-probability locations elicit an early Pd component within the P1 time window, indicating that a suppression effect is formed at the perceptual level [38,56]. However, our results consistently align with studies on the reward history, showing differences in the N2pc time window [39,40,41]. Anderson [19] found that the reward history exerts its influence on information processing at the level of competition for attentional selection, rather than amplifying feedforward processes in the early stages of perception, as the reward history affects the intensity of attentional orientation but not the speed [39,55]. Second, the statistical learning and the reward history influence the N2pc in the opposite effect direction. Our results align with previous findings, revealing that statistical learning for distractors results in a smaller N2pc component when the distractor is located at a high-probability location compared to low-probability locations, indicating a suppression effect based on the location probability. In contrast, the reward history leads to a larger N2pc for high-reward distractors compared to no-reward or low-reward distractors, reflecting a value-induced attentional capture effect.

Third, statistical learning and the reward history are independent sources for the attentional priority map and impact attention through different mechanisms. Statistical learning can develop a habit-based attention guidance mechanism through instrumental conditioning (i.e., participants are more likely to repeat orienting behaviors that have been reinforced [42]), exhibiting a proactive suppression mechanism, as the results showed a larger Pd for the distractors in the high-probability location. Previous studies have shown that the suppression of distractors is typically accompanied by Pd. By using an additional singleton task, Wang [38] found that only stimuli for distractors in high-probability locations elicited the Pd component (100–200 ms), suggesting that the suppression of distractor locations began at the early perceptual stage [56]. This suppression effect, occurring before the first attentional selection, is referred to as proactive suppression. The regulation of the spatial priority map effectively explains how distractor statistical learning forms a preemptive suppression effect. When a location is more likely to contain distractor information, its weight is downregulated, leading to suppression before attention is captured by the distractors [7,8,9,12,38]. For example, Kim [57] combined the additional singleton paradigm with eye tracking. The results showed that fewer initial fixations and saccades were slower to initiate when distractors were in high-probability locations compared to low-probability locations. This suggests that attentional priority accumulates more slowly for the high-probability location, and the priority weight of this location is lower than for all other locations in the visual field.

This downregulation of the spatial location weight results in a more generalized suppression effect. As we found, both the distractor and the target elicited the same degree of the Pd component at the high-probability location, indicating that the suppression effect is not specific to the distractors but is location-based. Consistent with previous research on distractor statistical learning, the continuous learning of the location-related information of a color singleton leads to the downregulation of the weight in the spatial priority map, which persists throughout the experiment, even when the target appears at that location.

The reward history, primarily learned through Pavlovian associative learning [20], exhibits an attentional capture mechanism for reward-related distractors, as evidenced by the larger reward-evoked N2pc. When the (repetitive) association between neutral stimuli and rewards is established, reward-related stimuli become more attractive and salient, similar to the incentive salience hypothesis [58,59], in which rewards make neutral stimuli more salient within the visual system. This suggests that reward distractors are more likely to be selected than non-reward distractors, as our results revealed a larger N2pc for reward distractors. This is consistent with the results of Qi [41]. In their study, during the practice phase, participants were trained to identify targets defined by two specific colors, receiving reward/no-reward feedback based on the target color. In the test phase, the previous reward color appeared on distractors in certain trials (with the target being a single shape). The N2pc component was observed only when the reward distractors appeared at the lateral position and the target was positioned vertically, but not for other non-target (neutral) stimuli. This reflects the increased attentional priority for reward-related features due to the reward history, leading participants to be more inclined to select previous reward features during the test phase. This priority enhancement was well demonstrated in an eye-tracking study [47]. Pearson used variants of an additional singleton task, simultaneously presenting both high- and low-reward distractors in certain trials to observe the effects of different values. The results showed that participants looked at the high-value distractors more often than the low-value distractors, indicating that high-reward distractors led to more oculomotor capture. This indicates that high-reward distractors have higher priority compared to low-reward distractors on a common saccadic priority map, leading to a higher frequency of saccades to the distractors in high-value reward trials compared to low-value reward trials.

In our study, the singleton clearly appeared as a distractor, and participants were encouraged to ignore it. However, we still observed that distractors under different reward conditions influenced target searching. The current results do not support the goal-driven attention selection strategy [1]. Furthermore, although different distractor colors were associated with different rewards, participants’ optimal strategy was to ignore the distractor color and focus on finding the target. We also found that reward distractors resulted in greater attentional capture compared to non-reward distractors. This contrasts the stimulus-driven attention mechanism [1]. We balanced the color salience of the distractors with different rewards between subjects, so the attentional capture effects from rewards were unlikely to be due to salience.

Although the current statistical learning is based on the spatial location and reward history regarding feature attributes, this dimensional difference may be a potential factor in their observed independent effects. Previous research has suggested that the statistical learning of distractor locations is a product of feature learning, with learning experiences from the feature dimension effectively influencing the regulation of spatial priority [7,9,43]. This indicates that spatial and feature dimensions are not entirely separate, and the encoding of feature dimension information can be linked to the automatic encoding of spatial information. Nevertheless, future research needs to further explore how statistical learning and the reward history within the same dimension independently affect attentional processing through cognitive processes.

In addition, the interference caused by the non-reward conditions in the attention capture effects (where N2pc was found only in low-probability locations) may not solely arise from the bottom-up capture by a color singleton in low-probability locations. We should also consider another potential source, such as the automatic capture effects induced by spatial vertical symmetry [60,61,62,63]. Previous research has shown a preference for mirror-symmetric objects along the y-axis, which is associated with increased activation in the ventral temporal cortex [63]. When the axis of symmetry is located at the center of the observer’s visual field, the recognition process is facilitated [60,62]. Since our EEG analysis for low-probability locations primarily focused on the other side of the horizontal position, and given that participants suppress high-probability locations, this may lead to attention capture by symmetric positions. Moreover, such simple symmetric visual stimuli typically do not require volitional effort and can be processed automatically [60].

This study had some limitations. Firstly, the sample size was relatively small. Although significant results were obtained, indicating a degree of robustness, the limited sample size may affect the generalizability of the findings [64]. A smaller sample size might lead to the observed effects less stable or reliable when applied to a broader population [64]. Future research should consider using larger samples to validate and extend our findings, thereby providing a more comprehensive understanding and ensuring that the results are generalizable across different populations. Secondly, the task used in this study was specific, aiming to investigate the independent effects of statistical learning and the reward history on attentional searching. While this controlled task design helped to reduce interference from external variables and improved the internal validity, it may limit the external validity of the findings [65]. The mechanisms of statistical learning and the reward history revealed by the experimental tasks may not fully reflect how these mechanisms operate in real-world situations. For example, real-world environments are often more complex and dynamic, involving a multitude of interacting variables that could influence how statistical learning and the reward history affect attention [65]. Therefore, while our findings reveal the independent effects of statistical learning and the reward history, future research should explore these processes in more complex and varied contexts to better understand how they interact and influence behavior in real-world situations. Finally, another potential limitation is the reliance on ERP measurements alone. While ERPs provide a high temporal resolution and insights into the temporal sequences of cognitive processes [66], they do not offer detailed spatial information [67]. Future studies might benefit from integrating ERP data with other neuroimaging techniques (such as fMRI or MEG) to provide a more comprehensive understanding of the neural mechanisms underlying the observed effects.

## 6. Conclusions

In summary, the present study demonstrates that reward history generates greater attentional capture effects by increasing the salience level of reward-related stimuli. Meanwhile, statistical learning produces greater attentional suppression effects in the earlier phases of the search by downregulating the spatial priority weights for specific locations. The enhancement of attentional capture for stimuli, whether in reward or no-reward conditions, did not vary across high- and low probability locations, as there was no interaction effect observed in either the RT or N2pc results. We also observed that statistical learning and reward history have distinct temporal and directional influences on cognitive processing. Taken together, this reflects that statistical learning and the reward history are independent resources for a common attentional priority map.

## Data Availability

The raw data supporting the conclusions of this article will be made available by the authors on request.
